# Comparison of endothelial function improvement estimated with reactive hyperemia index between ramipril and telmisartan in hypertensive patients

**DOI:** 10.1186/s40885-016-0060-y

**Published:** 2017-02-15

**Authors:** You-Jeong Ki, Jae-Bin Seo, Hack-Lyoung Kim, Woo-Hyun Lim, Hye Yeon Seo, Jin Yong Lee, Woo-Young Chung

**Affiliations:** 1 Department of Internal Medicine, Seoul Metropolitan Government Boramae Medical Center, Seoul, South Korea; 2 Seoul National University, College of Medicine, Seoul, South Korea

**Keywords:** Ramipril, Telmisartan, Endothelial function, Reactive hyperemia index, Pulse pressure

## Abstract

**Background:**

Endothelium has a function to regulate vascular tone by releasing mediators either vasodilating or vasoconstricting blood vessels. Endothelial dysfunction can be measured conveniently by Reactive Hyperemia Index (RHI) with a peripheral arterial tonometry. Angiotensin-converting enzyme inhibitors (ACEIs) and angiotensin II (AT II) receptor blockers (ARBs) are considered to have beneficial effects on endothelium through inhibition of AT II. This study was performed to compare the effect of ACEIs or ARBs on endothelial function estimated by RHI in hypertensive patients.

**Methods:**

Twenty consecutive patients with hypertension (57.9 ± 11.3 years, 60% men) were assigned to receive treatment with ramipril or telmisartan for eight weeks (*n =* 10 per group). Blood pressure (BP) and RHI were measured at baseline and after eight weeks treatment.

**Results:**

The two groups were similar in terms of demographic and laboratory characteristics. But baseline systolic BP and pulse pressure (PP) were higher in telmisartan group than ramipril group (systolic BP, 159 ± 6.83 vs 150 ± 7.49, *p =* 0.028; PP, 75.0 ± 14.0 vs 60.3 ± 12.4, *p =* 0.034). In both groups, systolic and diastolic BP decreased significantly after eight weeks treatment (p < 0.05 for each). Although PP reduced in both group (ramipril group, 60.3 ± 12.4 mm Hg to 50.4 ± 7.60 mm Hg; telmisartan group, 75.0 ± 14.0 mm Hg to 57.4 ± 15.1 mm Hg), change was statistically remarkable only in telmisartan group. During eight weeks, there was no significant changes of RHI in both groups. There was a positive relationship between decrease of PP after 8 weeks and the improvement of endothelial function only in ramipril group, but not in telmisartan group (ramipril group, *r* = 0.671, *p =* 0.034; telmisartan group, *r* = −0.487, *p =* 0.153).

**Conclusions:**

Despite PP reduction effect favoring endothelial function, it’s not correlated with RHI improvement with telmisartan. These findings suggest telmisartan itself may negatively influence endothelium dependent vasodilatation different from ramipril.

## Background

Vascular endothelium is a layer of cells lining the inner surface of vessels, separating the vascular wall from the blood [1]. Endothelium is a paracrine, endocrine and autocrine organ that is essential for vascular homeostasis and regulation of vascular tone [1]. Endothelium has a function to regulate vascular tone by release of vasodilator and vasoconstrictor substance [2]. Healthy endothelium has inhibitory effects on platelet aggregation and adhesion, smooth muscle cell proliferation and leukocyte adhesion [3].

An imbalance between endothelium derived relaxing factor and endothelin yields vascular tone dysfunction [2]. Endothelial dysfunction is an initiating event in atherosclerotic process and leads to plaque vulnerability, plaque rupture and thrombus formation [3, 4]. Thus the presence of endothelial dysfunction is a predictor of future cardiovascular events [5].

Endothelial function can be measured by Reactive Hyperemia Index (RHI) with a peripheral arterial tonometry (PAT) and flow medicated dilatation (FMD) of brachial artery with brachial artery ultrasound scanning (BAUS) [6, 7]. Both PAT and BAUS are validated method for evaluating peripheral endothelial function. Especially PAT is operator non-dependent, the most convenient and non-invasive method. The value of PAT has been validated in many clinical studies [7, 8]. Many studies have investigated the effect of AT II receptor blockers (ARBs) on endothelial function assessed by FMD. In a meta-analysis showed that ARBs improved the endothelial function compared with other antihypertensive agents and there was no difference between ARBs and angiotensin-converting enzyme inhibitors (ACEIs) [9].

**Fig. 1:**
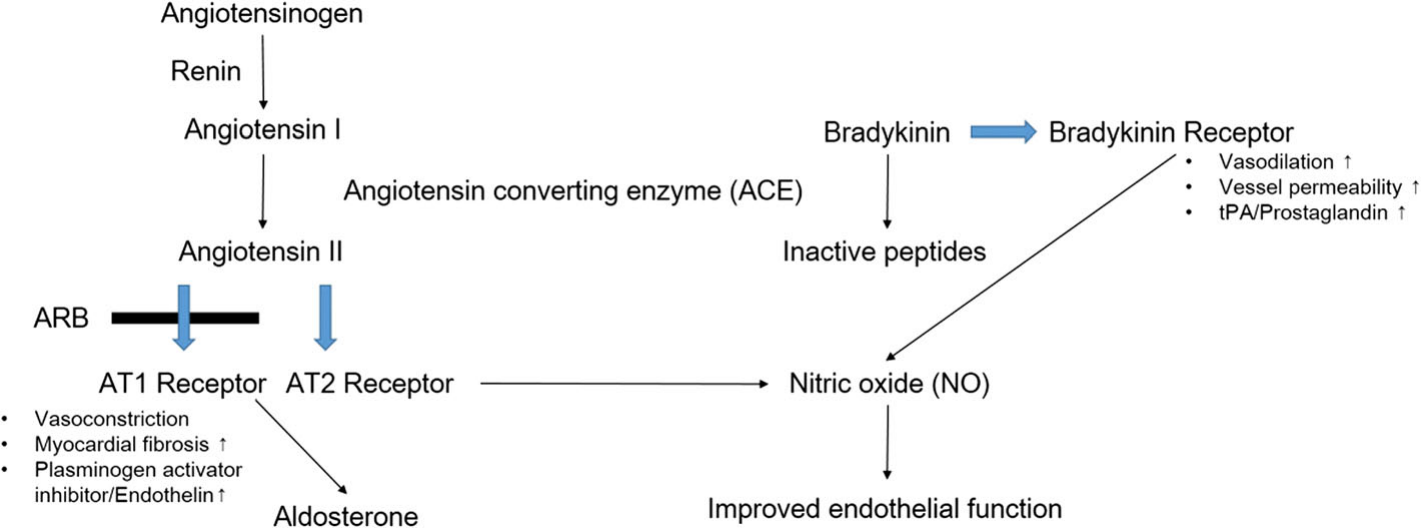
Angiotensin and kinin cascades affecting nitric oxide production ARB angiotensin II receptor blocker, AT1 angiotensin type 1, AT2 angiotensin type 2, tPA tissue plasminogen activator

Angiotensin II (AT II) regulates endothelial function and promotes endothelial cell apoptosis via reactive oxygen species (ROS) and oxidized low-density lipoprotein (oxLDL) uptake [10]. In many studies, ACEIs and ARBs have beneficial effects on endothelium through inhibition of AT II [3, 10]. Especially, ACEIs could improve vasodilation via decreased levels of AT II, increased levels of nitric oxide (NO) and bradykinin, whereas ARBs does not elevate bradykinin level [3]. In one aspect where ARBs can block AT II action of, ARBs seems better in protecting endothelial function. In the other aspect where ACE can booster bradykinin-NO pathway, ACEIs might be better for endothelial health (Fig. 1) [11].

However, comparison of endothelial function improvement between ACEIs and ARBs has not been well studied. This study was performed to compare the effect of ACEIs or ARBs on peripheral endothelial function estimated with RHI in hypertensive patients.

## Methods

### Study population

This single center study was performed at Boramae Medical Center (Seoul, Korea). From January 2014 to February 2016, a total of 24 consecutive subjects with essential hypertension, aged between 35 and 80 years were enrolled. Additional inclusion criteria were: patients already receiving hypertensive therapy or previous untreated hypertension patients. Hypertension was defined as systolic blood pressure (BP) ≥140 mm Hg or diastolic BP ≥90 mm Hg or daytime systolic BP ≥135 mm Hg or daytime diastolic BP ≥85 mm Hg at 24 h BP monitoring or nighttime systolic BP ≥125 mm Hg or nighttime diastolic BP ≥75 mm Hg at 24 h BP monitoring.

Subjects with following conditions were excluded: 1) patients of serum creatinine ≥1.5 mg/dL or during renal replacement therapy, 2) decompensated congestive heart failure, 3) systolic BP >180 mm Hg and diastolic BP >110 mm Hg, 4) acute coronary syndrome, 5) atrial fibrillation, 6) patients already receiving three-hypertensive therapy; high risk for the development of hypertensive catastrophe by cessation of medication 7) during pregnancy or planned for pregnancy subjects.

The participants were randomly assigned in a 1:1 ratio to ramipril group or telmisartan group according to a random number, generated by a web based randomization program of Medical Research Collaboration Center (MRCC) web site (https://mrcc.snuh.org/) of our institute. The number of study patients was decided to be 24 based on the size of research fund granted by the Korean Society of Hypertension. Patients were assigned to receive ramipril 5 mg once a day or telmisartan 40 mg once a day initially. Patients already taking antihypertensive treatment discontinued previous medications during two weeks for washout. Follow-up visits occurred at four weeks and eight weeks. If BP does not reach the target BP at four weeks, upward double dose titration of drugs was prescribed to reach the target BP of less than both <140 mm Hg systolic BP and <90 mm Hg diastolic BP. And medication switched from ramipril to telmisartan, when patients complained of ACEIs induced cough.

Data were collected at three time point: Baseline (BP, PTA, inflammatory marker, lipid panel, renal panel, HbA1c and fasting serum glucose), first follow-up visit at four weeks (BP and renal panel), second follow-up visit at eight weeks (BP, PTA, inflammatory marker, lipid panel, renal panel, HbA1c and fasting serum glucose).

The study protocol was approved by the Boramae Medical Center Human Research Ethics Committee. The protocol of this study was approved by the Institutional Review Board of Boramae Medical Center. And the trial was undertaken in accordance with the Declaration of Helsinki. Written informed consent was obtained from each patient at enrollment.

### Methods for endothelial function measurement

**Fig. 2:**
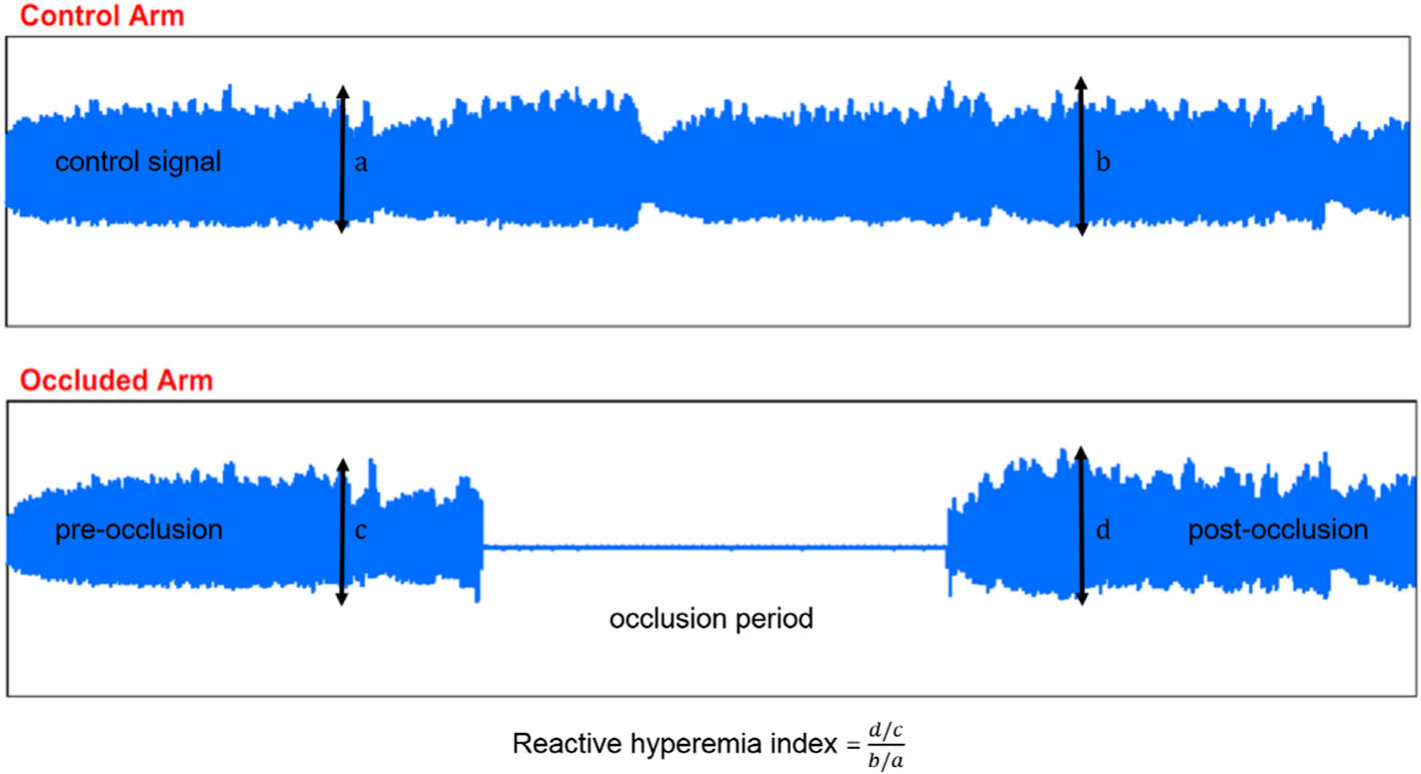
A representative reactive hyperemia arterial tonometry recording of a subject with normal reactive hyperemia index

All patients in each group underwent endothelial function test with PAT before the treatment and after 8 weeks treatment, respectively. The PAT (Endo-PAT2000; Itamar Medical Ltd., Caesarea, Israel) was used for non-invasive measurement of endothelial function. This system is a noninvasive technology that capture a beat-to-beat plethysmographic recording of the finger arterial pulse wave analysis (PWA) with pneumatic probes [7]. PWA was performed in accordance with a protocol that described by Bonetti PO [4]. Caffeine and alcohol are not allowed before PWA measurement. Each subject rested at sitting position more than 20 min in a quiet and thermoneutral (21 °C-24 °C) room when patients exposed cold or warm temperature before testing. Pneumatic BP cuffs were wrapped on study arm, while the other arm determined as control arm. The PAT finger probes were placed on the index finger of both hands. The reactive hyperemia procedure includes five minutes baseline recording, five minutes of blood flow occlusion through BP cuff inflation above systolic pressure and five minutes of recording after cuff deflation (Fig. 2).

RHI was calculated from the ratio of the average PWA during post-occlusive period compared with the average PWA during pre-occlusion baseline period. The ratio was normalized to the ratio from contralateral arm. Reproducibility of PAT was previously described by Bonetti PO [4].

### Statistical analysis

All numeric data are expressed as mean ± standard deviation for continuous variables or percentage for discrete variables. The differences in clinical characteristics and RHI were compared using the Wilcoxon-Mann–Whitney test for continuous variable and Chi-square test and Fisher’s exact test for discrete variables. Univariate associations between RHI and systolic BP, diastolic BP, pulse pressure (PP) and laboratory findings including lipid profile, HbA1c and inflammatory marker were evaluated using Pearson’s bivariate correlation analysis. Scatter plots were used to show the association between the 2 continuous parameters. The significance systolic BP, diastolic BP, PP and RHI changes were examined with Wilcoxon signed-rank tests for each group. A *P* value of <0.05 is used to indicate statistical significance. All statistical tests were performed with SPSS for Windows version 22 (IBM Co., Armonk, NY, USA).

## Results

Twenty-four Eligible patients were randomized from January, 2014 to February, 2016. Of the 24 patients who entered the study, 4 patients were excluded from the analysis due to follow up loss. Therefore a total of 20 patients (83.3%) entered the analysis. There were 12 male and 8 female, ranging from 37 to 75 years in age. 20 consecutive patients were assigned to receive treatment with ramipril or telmisartan (*n =* 10 per group). Baseline characteristics of this study population are shown in Table 1. The two groups were similar in terms of demographic and baseline laboratory characteristics. But baseline systolic BP and PP were higher in telmisartan group than ramipril group (systolic BP, 159 ± 6.83 vs 151 ± 7.49, *p =* 0.028; PP, 75.0 ± 14.0 vs 60.3 ± 12.4, *p =* 0.034).

**Table 1:** Baseline characteristics of study subjects

Characteristic	Total	Ramipril (*n =* 10)	Telmisartan (*n =* 10)	*P* value
Age, years	57.9 ± 11.3	55.9 ± 11.2	59.8 ± 11.7	0.449
Male, n (%)	12 (60%)	8 (80%)	4 (40%)	0.17
Traditional risk factors				
Diabetes mellitus, n (%)	5 (25%)	2 (20%)	3 (30%)	1.0
Current smoking, n (%)	5 (25%)	4 (40%)	1 (10%)	0.303
Systolic blood pressure, mmHg	155 ± 8.04	151 ± 7.49	159 ± 6.83	0.028
Diastolic blood pressure, mmHg	87.2 ± 12.0	90.6 ± 9.00	83.7 ± 14.0	0.08
Pulse pressure, mmHg	67.7 ± 14.9	60.3 ± 12.4	75.0 ± 14.0	0.034
Mean blood pressure, mmHg	110 ± 8.25	111 ± 6.21	109 ± 10.1	0.518
Major laboratory findings				
Fasting blood glucose, mg/dL	119 ± 44.2	104 ± 9.86	128 ± 55.1	0.77
HbA1c, %	5.93 ± 0.68	5.70 ± 0.62	6.6	0.18
Total cholesterol, mg/dL	185 ± 40.0	186 ± 43.2	184 ± 40.1	0.954
HDL cholesterol, mg/dL	47.7 ± 9.26	47.3 ± 9.37	48 ± 10.0	0.936
LDL cholesterol, mg/dL	113 ± 32.3	104 ± 32.3	122 ± 32.7	0.423
Triglyceride, mg/dL	180 ± 101	163 ± 93.2	197 ± 114	0.521
Potassium, mmol/L	4.31 ± 0.294	4.41 ± 0.219	4.16 ± 0.344	0.135
hs-CRP, mg/dL	0.433 ± 0.825	0.085 ± 0.06	1.13 ± 1.27	0.364
Reactive hyperemia index	1.87 ± 0.273	1.94 ± 0.284	1.80 ± 0.258	0.272

*HDL* high-density lipoprotein, *LDL* low-density lipoprotein, hs-*CRP* high sensitive C-reactive protein

**Fig. 3:**
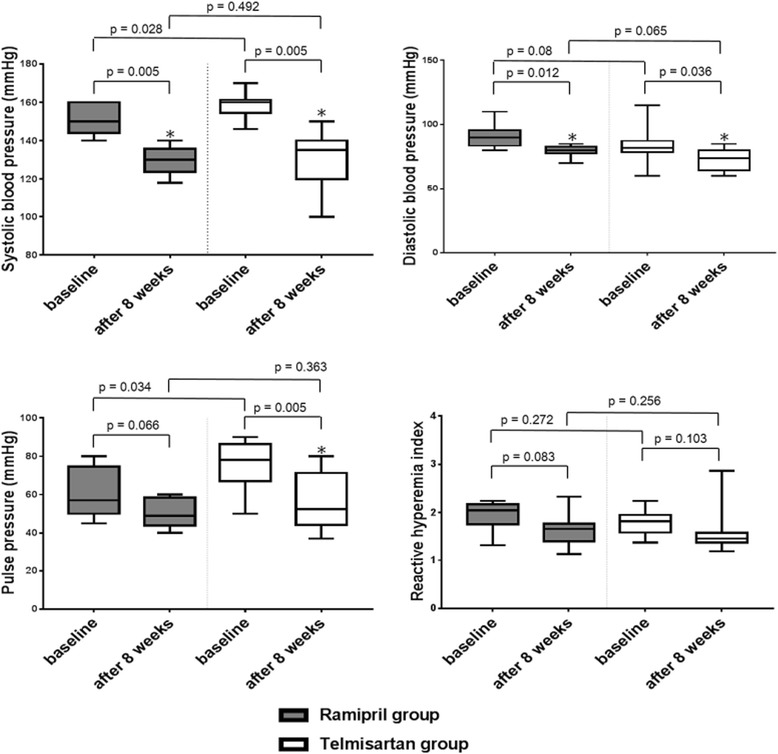
Systolic BP, diastolic BP, PP and reactive hyperemia index in patients at baseline and after 8 weeks of treatment with ramipril or telmisartan ∗ p < 0.05 vs baseline

Figure 3 and Table 2 show BP, PP and their changes during the study period. In both groups, systolic and diastolic BP decreased significantly after 8 weeks treatment. The systolic BP ranged from 140 to 170 mm Hg and the diastolic BP ranged from 60 to 115 mm Hg at baseline. The BP was 151 ± 7/91 ± 9 mm Hg in the ramipril group and 159 ± 7/84 ± 14 mm Hg in the telmisartan group at baseline. At 8 weeks follow up, BP was 130 ± 7/80 ± 5 mm Hg in ramipril group and 131 ± 14/73 ± 9 mm Hg in telmisartan group. BP reduction from baseline to the 8 weeks follow up was 21/11 mm Hg (systole/diastole) in the ramipril group and 28/11 mm Hg (systole/diastole) telmisartan group, respectively (p value, Wilcoxon signed-rank test : 0.005, 0.012, 0.005, 0.036, respectively). The target BP (<140 mm Hg systolic and <90 mm Hg diastolic) was achieved in 9 (90%) patients in the ramipril group and 6 (60%) in the telmisartan group at 8 weeks follow up. PP reduced in both group (ramipril group, 60 ± 12 mm Hg to 50 ± 8 mm Hg; telmisartan group, 75 ± 14 mm Hg to 57 ± 15 mm Hg). But, change was statistically remarkable in telmisartan group, while it was borderline significant in ramipril group (*p =* 0.066, Wilcoxon signed-rank test).

**Table 2:** Blood pressure and pulse pressure before and after treatment

Parameter	Systolic blood pressure			Diastolic blood pressure			Pulse pressure		
	Baseline	After 8 weeks	*P* value^b^	Baseline	After 8 weeks	*P* value^b^	Baseline	After 8 weeks	*P* value^b^
Total	155 ± 8.04	130 ± 11.1	<0.001	87.2 ± 12.0	76.2 ± 7.61	0.001	67.7 ± 14.9	53.9 ± 12.2	0.001
Ramipril 5 mg group	151 ± 7.49	130 ± 7.12	0.005	90.6 ± 9.00	79.2 ± 5.25	0.012	60.3 ± 12.4	50.4 ± 7.60	0.066
Telmisartan 40 mg group	159 ± 6.83	131 ± 14.4	0.005	83.7 ± 14.0	73.1 ± 8.61	0.036	75.0 ± 14.0	57.4 ± 15.1	0.005
*P* value^a^	0.028	0.492		0.08	0.065		0.034	0.363	

^a^Comparison between patients using ramipril and telmisartan

^b^Comparison between before and after treatment

Table 3 and Fig. 3 demonstrate the baseline and 8 weeks follow up RHI of both groups. The baseline RHI was 1.94 ± 0.28 in ramipril group and 1.80 ± 0.26 with telmisartan group (*p =* 0.272). At 8 weeks follow up, the RHI was 1.64 ± 0.33 in ramipril group and 1.59 ± 0.46 in telmisartan group (*p =* 0.256). During 8 weeks, there was no significant changes of RHI in both groups.

**Table 3:** Reactive hyperemia index before and after treatment

	Baseline	After 8 weeks	*P* value^b^
Total	1.87 ± 0.273	1.61 ± 0.393	0.014
Ramipril 5 mg group	1.94 ± 0.284	1.64 ± 0.331	0.083
Telmisartan 40 mg group	1.80 ± 0.258	1.59 ± 0.464	0.103
*P* value^a^	0.272	0.256	

^a^Comparison between patients using ramipril and telmisartan

^b^Comparison between before and after treatment

**Fig. 4:**
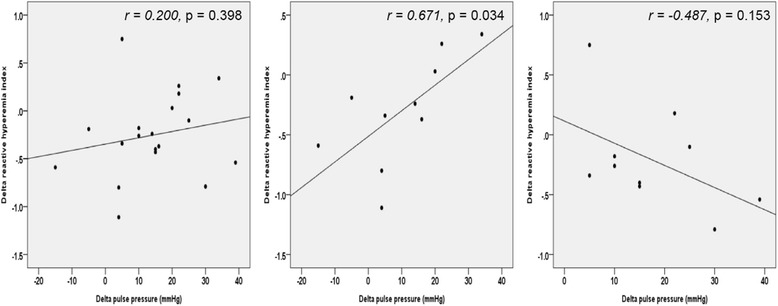
Association of delta pulse pressure and delta reactive hyperemia index in total patients (left), ramipril (middle) and telmisartan (right) group Delta reactive hyperemia index indicates RHI after 8 weeks minus RHI at baseline, and Delta PP Baseline PP minus PP after 8 weeks

As mentioned above, the effects of 8 weeks of treatment on PP were more marked and significant in telmisartan group. However, despite of this favorable change of PP, telmisartan group have shown a tendency of discrepancy between changes of PP and RHI, although statistically insignificant, which means aggravation of endothelial function even with decreased PP. On the other hand, there was a positive relationship between decrease of PP after 8 weeks and increase of RHI in ramipril group (ramipril group, *r* = 0.671, *p =* 0.034; telmisartan group, *r* = −0.487, *p =* 0.153, Fig. 4).

Both medications were relatively well tolerated except one adverse event. One patient in ramipril group experienced transient global amnesia probably due to small vessel infarction. Only one patient had to change her medication from ramipril to telmisartan because of dry cough caused by ramipril.

## Discussions

The present study intended to investigate if ramipril and telmisartan affect endothelial function differently from each other after the treatment for eight weeks in hypertensive patients. During short period of treatment, while endothelial function estimated with RHI did not change in both groups. Both agents reduced systolic and diastolic blood pressure effectively without difference between two groups.

But, the effects of 8 weeks of treatment on PP were more marked and significant in telmisartan group. Despite these favorable hemodynamic changes, telmisartan didn’t show improving endothelial function. Although mean RHI was stationary in ramipril group during the treatment, what extent RHI changed by is well positively correlated with changes of PP, which means the more decrease PP, the better endothelial function. However, this correlation was not seen in telmisartan group.

A few studies postulated wide PP adversely affect endothelial function [12–15]. Because any other factors associated with endothelial function such as lipid profile, smoking history, were not different between groups, the fact improvement of endothelial function is not correlated with decrease of PP in telmisartan group suggests telmisartan itself may negatively influence endothelium dependent vasodilatation.

Ramipril, a kind of ACEIs reduces the AT II level by inhibition of AT I converts to AT II, increases NO and other endothelial mediators induced by bradykinin via inhibition of kininase II, which is responsible for the degradation of bradykinin [16]. Bradykinin affects vasodilatation via NO, prostacyclin and hyperpolarizing factor [17].

Telmisartan, a kind of ARBs inhibits the renin angiotensin system (RAS) in different way from ACEIs. ARBs bind to the AT1 receptor, interrupting its activation. As a result of AT1 receptor blockade, ARBs increase AT II concentration by a positive feedback resulting in stimulating AT II type 2 receptors (AT2Rs) as alternative pathway. AT2Rs have a role in vasodilatation by counteracting AT1 receptor mediated vasoconstriction [18]. And ARBs lead to bradykinin-dependent NO release through AT2R [19]. AT II binding to AT2Rs induces intracellular acidification, which activates a kininogenase. This increases production of bradykinin, which increases NO and vascular smooth muscle cell relaxation [10, 19]. But, many studies have demonstrated negative effects of persistent AT2R stimulation recently. AT2Rs mediate arterial hypertrophy, cardiac hypertrophy and fibrosis and anti-angiogenic effect on cardiovascular tissues, although it’s uncertain if all of these changes accompany endothelial dysfunction [18, 20, 21].

In this respect, ‘ARB-acute myocardial infarction (MI) paradox’ was issued in 2004 after releasing VALUE trial. This theory focused on increase of MI in valsartan arm compared with amlodipine arm in the VALUE trial (hazard ratio = 1.19, 95% CI 1.02 to 1.38, *p =* 0.02) [22, 23]. Other meta-analysis of ACEIs and ARBs trials showed that ACEIs (versus placebo, non ARBs comparator and ARBs) reduced the relative risk of MI by 14% (OR 0.86, 95% CI 0.82 to 0.90, p < 0.001), whereas ARBs (versus placebo, non ACEIs comparator and ACEIs) increased the risk of MI (OR 1.08, 95% CI 1.01 to 1.16, *p =* 0.03) [24]. Although a meta-analysis of Tsuyuki and McDonald reported that ARBs did not increase the risk of MI with OR of 1.03 (95% CI 0.93 to 1.13) compared to ACEIs, many subsequent studies disproved the results [25]. The BPLTTC meta-regression analysis of 26 trials (17 trials with ACEIs and 9 trials with ARBs) showed that both ACEIs and ARBs have BP dependent risk reduction of stroke, coronary heart disease and heart failure [26]. However, after adjusted for BP reduction within trials, the estimated risk reduction for coronary heart disease was 9% (3 to 14%, *p =* 0.004), whereas ARBs do not. Furthermore, ONTARGET compared telmisartan with ramipril in patients with vascular disease or high risk diabetes [27]. Telmisartan bad better BP lowering effect than ramipril, but increased MI by 7%, although statistically insignificant.

This randomized trial showed patients in both groups didn’t show improvement of endothelial function than baseline after 8 week treatment, even though blood pressure reduction was enough. This finding the improvement of endothelial function and reduction of BP are not corresponding has ever been reported by Ghiadoni et al. [28]. In his study, ACEIs, calcium antagonists, ARBs, and beta blockers similarly reduced BP, but only perindopril, one type of ACEI, improved endothelial function.

It’s notable the reduction of PP is well correlated with the improvement of endothelial function expressed as a RHI not in telmisartan group, but in ramipril group. Higher PP exerts lower shear stress against arterial wall subsequently resulting in larger oxidative stress and reduced NO production [13]. In telmisartan group, PP decreased significantly after treatment. However, PP reduction was not related with endothelial function improvement in telmisartan group. This finding suggests telmisartan is likely to have a prohibiting effect on endothelial functional recovery initiated by PP reduction.

Several limitations of our study should be mentioned. First, the present study is too small study to make a confirmatory conclusion regarding inferiority of telmisartan in the aspect of endothelial function. Actually, there are many studies revealing favorable effects of ARBs on endothelial function. Shuang Li et al. investigated 1737 patients of 22 trials with endothelial dysfunction, and showed that ARBs could improve endothelial function assessed by FMD compared with placebo or other hypertensive medication such as calcium channel blockers, beta-blockers and diuretics [9]. But, ARBs have no significant difference with ACEIs. Other many studies show ARBs and ACEIs improved endothelial function without difference. Hornig et al. demonstrated that ACEIs and ARBs improved endothelial vasodilatation to a similar extent in coronary artery disease patients through increasing NO availability [29]. Second, blood flow in cutaneous vessels is known to depend on sympathetic and autonomic nervous systems. Therefore, it is possible that changes in the finger RHI may occur due to changes in room temperature and mental status. We attempted to control these confounders by studying in a quiet and automatically thermostatic room and adjusting pulse amplitude of study arm with one of the contralateral control arm as a reference. Third, we just investigated short term effects of ACEIs and ARBs. It should be further evaluated the results shown in this study can be consistent in long term aspect.

## Conclusion

In hypertensive patients, ramipril and telmisartan reduced systolic and diastolic BP after eight weeks treatment. But, PP lowering effect was shown statistically significant only in telmisartan group. Despite PP lowering effect, telmisartan didn’t show endothelial function improvement proportional to reduced PP. These findings suggest telmisartan may negatively influence endothelium and emphasize the role of bradykinin mediated NO production pathway in the control of endothelial function, which is saved with ramipril treatment.

## Abbreviations

ACEIs: Angiotensin-converting enzyme inhibitors
ARBs: Angiotensin II receptor blockers
AT II: Angiotensin II
AT2R: Angiotensin II type 2 receptor
NO: Nitric oxide
PAT: Peripheral arterial tonometry
PWA: Pulse wave analysis
RAS: Renin angiotensin system
RHI: Reactive hyperemia index
